# Good Outcome Following Copeland Hemiarthroplasty for Acromegalic Arthropathy

**DOI:** 10.1155/2011/814073

**Published:** 2011-12-22

**Authors:** S. E. Johnson-Lynn, J. Candal-Couto

**Affiliations:** Department of Orthopaedic Surgery, Wansbeck General Hospital, Woodhorn Lane, Northumberland, Ashington NE63 9JJ, UK

## Abstract

We report the unusual case of a patient with acromegalic arthropathy who presented with severe shoulder arthrosis with marked osteophytosis. This patient is currently pain-free and has good shoulder function 4 years following a Copeland shoulder hemiarthroplasty. Acromegaly is a rare condition of growth hormone oversecretion, but arthropathy is a common feature of the natural history of the disease. This is the first published case report of the outcome of shoulder arthroplasty in a patient with acromegalic arthropathy and demonstrated that a good result can be obtained in this patient.

## 1. Case Report

The patient was diagnosed with acromegaly in 1980 and was treated with transsphenoidal surgery and radiotherapy. These treatment modalities were unsuccessful in controlling growth hormone levels. Control was gained many years later by bromocriptine treatment combined with the delayed effect of radiation.

**Figure 1:**
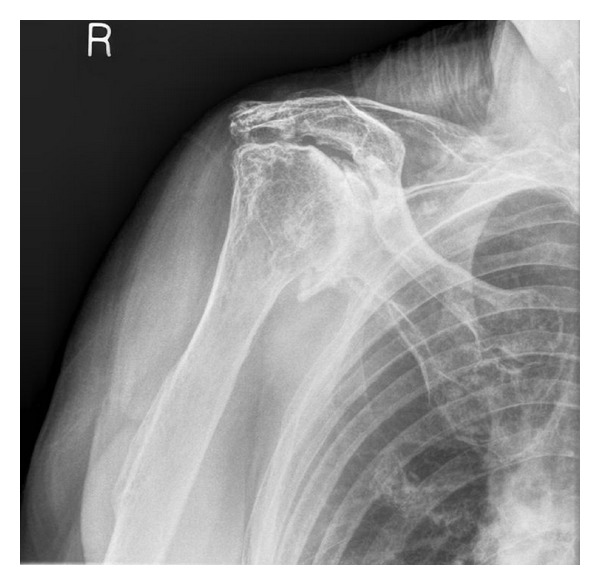
AP radiograph of the right shoulder taken preoperatively.

**Figure 2:**
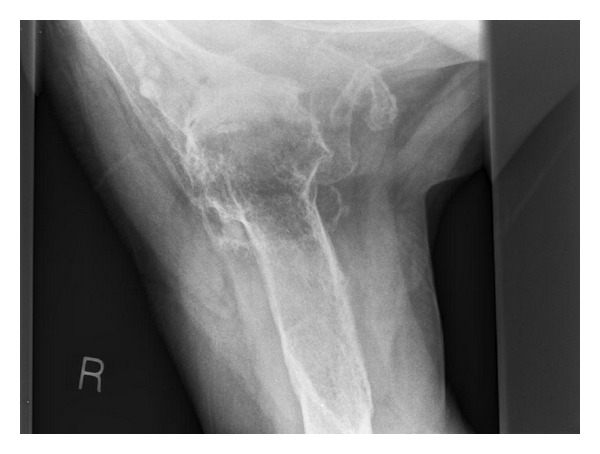
Axillary lateral radiograph of the right shoulder taken pre-operatively.

A fall in 2005 resulted in an olecranon fracture requiring open reduction and internal fixation, and during followup the patient complained of severe pain and stiffness of the right shoulder. The patient reported an inability to perform any active movements of the shoulder, and passive range of movement was restricted to 40 degrees of elevation, 20 degrees of external rotation, and internal rotation to the coronal midline. X-rays revealed severe arthrosis with extensive osteophytosis (Figures 1 and 2).

A right Copeland hemiarthroplasty was performed in 2006, and multiple osteophytes were removed. The procedure was technically challenging, with both a deltopectoral and a Mackenzie anterosuperior approach being required in order to resect the extensive osteophytes.

**Figure 3:**
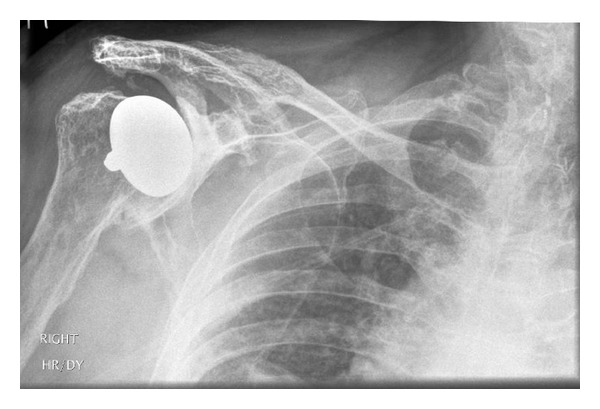
AP radiograph of the right shoulder taken 4 years postoperatively.

**Figure 4:**
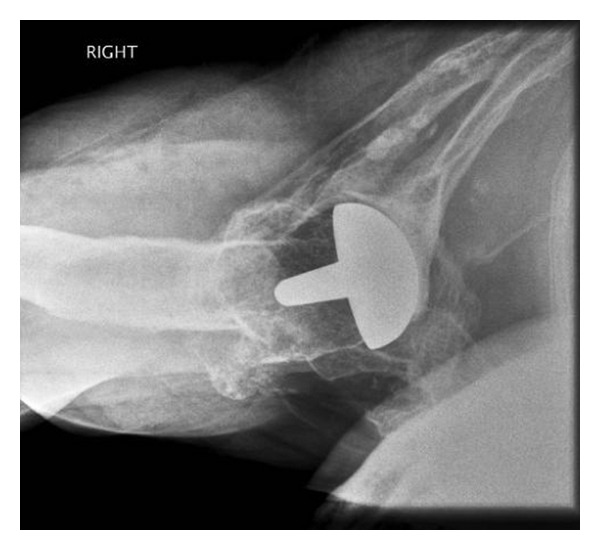
Axillary lateral radiograph of the right shoulder taken 4 years postoperatively.

At two weeks postoperatively, an excellent recovery was noted, with a full passive range of movement and active elevation to 90 degrees achieved. By the second postoperative month, the patient was pain-free, active abduction, and elevation of 100 degrees was achieved with good power. At the latest followup, 4 years postoperatively, the patient has a pain-free shoulder and is easily able to perform activities above shoulder height. X-rays show no radiological evidence of loosening or bone resorption (Figures 3 and 4).

## 2. Discussion

Acromegaly is a rare disorder (annual incidence of 5 in a million) of excess growth hormone secretion by a pituitary adenoma of somatotroph cells. Growth hormone excess has effects on multiple systems; it's most common musculoskeletal effects are gigantism, prognathism, arthropathy, osteopaenia, carpal tunnel syndrome, [1] hypermobility, spondylarthropathy, and fibromyalgia [2, 3].

Acromegalic arthropathy most commonly affects knees, hips, and shoulders. [4] 50–60% acromegalic patients have pain affecting the axial skeleton or one or more large peripheral joints at presentation [2, 5]. Early in the course of the disease, reversible changes are caused by elevated growth hormone and IGF-1 levels. These include growth of cartilage causing a congested joint and the radiological appearances of increased joint space. As the disease progresses, altered joint mechanics result in intra-articular trauma, triggering the characteristic severe osteophyte formation [5, 6]. At this point, the process is irreversible. Long-term control of growth hormone levels may lead to a reduction in arthritis [7].

To our knowledge, this case study is the first to present the outcome of a hemiarthroplasty as successful treatment for acromegalic arthropathy of the shoulder. The outcome in this case is particularly interesting as the patient had active acromegaly at the time of surgery, and the acromegalic arthropathy was actively progressing with further osteophytosis.

## 3. Conclusion

This is the first published case report of the outcome of shoulder arthroplasty in a patient with acromegalic arthropathy and demonstrated that a good result can be achieved in this patient in terms of pain relief and range of movement.
